# Individual and environmental risk factors associated with fecal glucocorticoid metabolite concentrations in zoo-housed Asian and African elephants

**DOI:** 10.1371/journal.pone.0217326

**Published:** 2019-09-04

**Authors:** Janine L. Brown, Kathy Carlstead, Jessica D. Bray, David Dickey, Charlotte Farin, Kimberly Ange-van Heugten

**Affiliations:** 1 Center for Species Survival, Smithsonian Conservation Biology Institute, Front Royal, Virginia, United States of America; 2 Department of Animal Science, North Carolina State University, Raleigh, North Carolina, United States of America; 3 Department of Statistics, North Carolina State University, Raleigh, North Carolina, United States of America; Humboldt-Universitat zu Berlin, GERMANY

## Abstract

A recent large-scale welfare study in North America involving 106 Asian (*Elephas maximus*) and 131 African (*Loxodonta africana*) elephants at 64 accredited facilities identified links (i.e., risk factors) between zoo environmental factors and a number of welfare outcomes (stereotypic behavior, ovarian acyclicity, hyperprolactinemia, walking and recumbence, body condition, health status, serum cortisol). For this population of elephants, we used the same epidemiological methods to examine associations between those risk factors and two additional welfare outcomes, mean concentration and individual variability (CV) of fecal glucocorticoid metabolite concentrations (FGM) as indicators of stress. Results indicate that African elephants are more responsive to social stressors than Asians, and that poor joint health is a stress-related welfare problem for Asian, but not African elephants in the North American population. For both species, higher FGM concentrations were associated with zoos located at more northern latitudes, whereas lower FGM concentrations were associated with having free access to indoor/outdoor spaces, and spending more time in managed interactions with staff. Also important for captive management, elephants having diverse enrichment options and belonging to compatible social groups exhibited reduced intra-individual variability in FGM concentrations. Our findings show that aspects of the zoo environment can be potential sources of stress for captive elephants, and that there are management activities that may facilitate coping with zoo conditions. Given species differences in factors that affected FGM, targeted, species-specific management approaches likely are needed to ensure good welfare for all elephants.

## Introduction

Modern zoos strive to ensure animals under human care experience a high standard of welfare that meets emotional and physical health needs [1]. Asian (*Elephas maximus*) and African (*Loxodonta africana*) elephants in zoos have received considerable scrutiny in the last two decades because of concerns over welfare and management practices [2]. To create sustainable captive populations, it is important that zoo animal programs evaluate the basic husbandry needs of individual animals, as well as the more complex factors that may affect welfare in a captive environment. For example, an earlier study of 112 female zoo-housed elephants in North America found a significant effect of “facility” on longitudinal serum cortisol concentrations, but no significant effect of “species” or “management” (i.e., free contact—elephants and people share the same space; or protected contact—elephants and people are separated by a barrier) [3], suggesting that facility-specific factors exist that may affect stress and welfare status in captive elephants.

A more recent Elephant Welfare Project (EWP) took an epidemiological approach to determine how factors in the zoo environment impact a number of welfare indicators in captive elephants. That study, conducted by a multi-institutional team of researchers, included 237 elephants at 64 Association of Zoos and Aquariums (AZA)-accredited zoos, and found a variety of factors correlated with welfare outcomes. In particular, enrichment (physical items and facility features) and social (herd composition and interactions) factors were important for normal pituitary-ovarian function [4] and reducing stereotypic behaviors [5]; diversity of feeding practices and exercise reduced the likelihood that an elephant would be overweight [6,7]; softer exhibit substrates were good for physical and behavioral health [8,9]; and positive keeper-elephant relationships were mutually beneficial [10]. Overall, environments that provided diversity and choice were of greater importance to elephant -welfare—than exhibit size alone [11]. A remaining question is if these factors also affect physiological stress responses in individual elephants.

The most commonly used bio-markers of stress and, by extension welfare, are glucocorticoids (GC) secreted from the adrenal cortex in response to a stressor [12,13]. The primary role of GCs is energy regulation and mobilization [14,15], but at higher concentrations they facilitate physiological changes associated with the stress response [14]. Stimuli both favorable and unfavorable to welfare can increase GC release; however, most studies of captive wildlife focus on how prolonged exposure to psychological or physical stressors increase GCs and may affect well-being, such as causing immunosuppression, decreased wound healing, increased susceptibility to disease, poor reproduction, and development of stereotypic behaviors [16]. Circulating GCs have been measured in elephants [3,17,18,19], although an important consideration is whether the act of collecting blood itself elicits a response [20,21]. For that reason, noninvasive measures of GCs or their metabolites excreted in feces (fecal glucocorticoid metabolites, FGM) have provided a robust tool for assessing welfare in wildlife species [22,23], including elephants [24,25,26,27,28,29,30,31,32].

The biological validity of FGM to monitor adrenal cortex activity has been demonstrated in elephants under a variety of conditions. Normal physiological increases in FGM are observed during parturition [17], in association with musth [29,33] and during the follicular phase of the estrous cycle [19]. Increases also occur in response to stressful conditions, such as negative interactions with humans and episodic loud noises [34], opening of a zoo to the public for the first time [35], work associated with logging [36,37], participating in public festivals and processions [29], being housed in small enclosures [38], construction [39], and in association with transportation and relocation [34,40,41]. More recently, Edwards et al. [42] found positive correlations between the number of clinical cases in the EWP study and the coefficient of variation (CV) for both serum cortisol and FGM, suggesting that within-individual variation in FGMs also may be an important welfare indicator. Thus, non-invasive glucocorticoid monitoring can be a powerful tool for assessing stress responses and welfare status, especially when combined with evaluations of health or behavior.

The goal of this study was to determine how previously identified risk factors associated with physical [7,9], behavioral [6,8,43], and physiological [4, 18] outcomes measured in the EWP to date affect FGM concentrations using the same epidemiological approach. We hypothesized that risk factors for ovarian acyclicity, hyperprolactinemia, obesity, stereotypy, poor foot and joint health, lower rates of physical activity or recumbence, and higher serum cortisol responsiveness are associated with higher FGM mean concentrations and variability. The ultimate goal is to better understand relationships between FGM and welfare outcomes, and how they are influenced by extrinsic forces—important information needed to optimize management of elephants in zoo settings.

## Materials and methods

### Ethics statement

This research was approved by the Animal Care and Use Committee of the Smithsonian National Zoo (NZP-ACUC #11/10).

### Study population and sample collection

The study consisted of 237 captive elephants, 106 Asian (85 females; 21 males) and 131 African (104 females; 27 males), housed at 64 American Zoo and Aquarium (AZA) accredited facilities throughout North America that participated in the EWP. Fresh fecal samples were collected by keepers at a frequency of every other week for 12 months. Samples were collected by keepers fresh from the ground in the morning within 2 hours of defecation, mixed to obtain homogeneity, and then 5–10 subaliquots (~50–100 g) placed into Whirlpak^®^ plastic bags, and frozen (-20°C) immediately. All fecal samples were collected at the same time as data for the other EWP studies, which was for 1 year in 2012 [4–9, 18, 42–43].

### Fecal extraction and GC metabolite analysis

Fecal samples were lyophilized (Labconco, Kansas City, MO), and 0.1 g (± 0.02) of well-mixed fecal powder was placed into 16 x 125 mm glass tubes (Fisher Scientific; Pittsburgh, PA). Five ml of 80% methanol was then added and the samples were mixed for 30 minutes on a multi-tube vortexer (Glas-Col; Terre Haute, IN), followed by centrifugation for 20 min at 2500 x g (Sorvall RC 3C Plus; Thermo Fisher Scientific, Waltham, MA). Each supernatant was recovered and the remaining pellet was re-suspended in 5 ml of 80% methanol and extracted again. The two supernatants were combined into a 16 x 125 mm glass tubes and dried under forced air in a fume hood overnight. Extracted samples were reconstituted in 1 ml of 100% methanol, dried again, and then buffer (1 ml, 0.149 M NaCl, 0.1 M NaPO_4_; with pH 7.0) added and the tubes sonicated (Part# 08895–60; Cole-Parmer, Vernon Hills, IL) for 30 seconds to dissolve particulates. Finally, all samples were diluted (1:8) in assay buffer (Cat. No. X065, Arbor Assays, Arbor, MI, USA) and stored at –20°C until enzyme immunoassay (EIA) analysis.

Concentrations of FGM were determined using a double-antibody enzyme EIA with a polyclonal rabbit anti-corticosterone antibody (CJM006) validated for elephants [32]. Standards (3.9–1000 pg/well; Sigma Diagnostics, St. Louis, MO), samples, and controls were added in duplicate (50 μl per well) to pre-coated goat anti-rabbit IgG, 96-well plates at room temperature. Corticosterone-horseradish peroxidase (25 μl, 1:20,000 dilution) was immediately added to all wells, followed by 25 μl anti-corticosterone antibody (1:60,000) that was added to all but non-specific binding wells. The plates were covered with microplate sealers and incubated at room temperature on an agitator (Model E6121; Eberbach Corp., Ann Arbor, MA) for 1 hour. All plates were then washed four times (1:20 dilution, 20X Wash Buffer Cat. No. X007; Arbor Assays), blotted dry, and 100 μl of TMB (3, 3', 5, 5'–tetramethylbenzidine) (Moss Inc., Pasadena, MD) was added. Plates were incubated for 30–45 min at room temperature without shaking, and the reaction stopped by adding 50 μL of a 1 N HCl solution. Optical density was read in a plate reader at 450 nm (OPsys MR; Dynex Technologies, Chantilly, VA). The inter-assay coefficient of variation (CV %) for the high control was 8.1%, and the low control CV% was 15.1% (n = 200 plates); intra-assay CV was <10% as all samples with duplicate CVs over 10% were reanalyzed. Assay sensitivity (based on 90% binding) was 0.14 ng/ml.

### Statistical analysis

Independent variables used for these analyses were chosen based on their significance as risk factors in already-published multi-variable models for other welfare indicators of the EWP: reproductive dysfunction as indicated by ovarian acyclicity and hyperprolactinemia [4], stereotypy [43], body condition [7], foot and joint health [9], walking distance and recumbency [6,8], and serum cortisol [18]. Full details regarding data collection and variable creation are provided in several EWP publications [5,11,44]. Table 1 summarizes the independent variables identified as significant “risk factors” for each welfare indicator and descriptions of each independent variable. Elephant-specific independent variables were: *Age*, *Sex*, *Percent Time in Mixed-Sex Herds*, *Social Group Contact*, *Walking Hours Per Week*, *Percent Time with Juveniles*, *Percent Time Housed Separately*, *Transfers*, *Percent Time In/Out Choice*, *Social Experience*, *Recumbence Rate*, *Percent Time on Hard Substrate*, *Percent Time on Soft Substrate*, *Space Experience Outdoors at Night*, *Space Experience with In/Out Choice*, *Joint Health*, *Space Experience Total at Night*, *Mean Daily Walking Distance*, *Mean Serum Cortisol*, *Elephant Positive Behaviors*, and *Elephant Interacts with Public*. Measured on a zoo-level were *Season*, *Enrichment Diversity*, *Alternative Feeding Methods*, *Feeding Diversity*, *Percent Time Managed*, *Keeper Positive Opinions of Elephants*, *Keeper as Herdmate* and *Latitude of Zoo*.

**Table 1:** Significant independent variables that were identified as risk factors for welfare outcomes for either or both species in published multi-variable models from the Elephant Welfare Project.

Welfare Indicators	Independent Variables^1^	Definition of independent variable
Ovarian acyclicity^2^	Percent Time in Mixed Sex Herds (*unpub*.)	Sum of monthly percent time spent in social groups where both males and females are present
	Age	Age of elephant in years in 2012
	Enrichment Diversity	Shannon diversity index score of enrichment activities types and frequencies conducted at zoo
Hyperprolactinemia^2^	Alternate Feeding Methods	The proportion of all feedings where food was presented in a foraging device, hidden, or hung above the exhibit
	Social Group Contact	Maximum number of unique social groups focal animal is part of
Body Condition^3^	Walking, Hours/Week	Number of reported hours spent walking elephants each week, ranging from 1 (< 1 hour per week) to 7 (14 or more hours per week)
	Feeding Diversity	Shannon diversity index score of feeding types and frequencies conducted at zoo
	Sex (ref: male)	Male or female
Daytime Stereotypy^4^	Percent Time Managed	Sum of percent time spent in activities managed by caretaking staff
	Percent Time with Juveniles	Sum of monthly percent time spent in social groups where an elephant 7 years or younger was present
	Percent Time Housed Separately	Sum of monthly percent time spent housed in a social group of one
	Transfers	Total number of inter-zoo transfers an elephant has experienced
Nighttime Stereotypy^4^	Percent Time In/Out Choice	Sum of monthly percent time spent in environments where there is a choice of indoors or outdoors
	Social Experience	The average weighted (by percent time) size of all social groups in which an elephant spent time
Recumbence^5^	Recumbence Rate	Hours recumbent per day, averaged over all days of data collection
	Percent Time on Hard Substrate	Sum of monthly percent time spent in environment with 100% concrete or stone aggregate substrate
	Percent Time Soft Substrate	Sum of monthly percent time spent in environment with 100% grass, sand, or rubber substrate
	Space Experience Outdoor Night (per 500 ft^2^)	The average weighted (by percent time) size of all environments in which an elephant spent time in outdoor environments only
	Percent Time Housed Separately	Sum of monthly percent time spent housed in a social group of one
Muscoskeletal Score^6^	Space Experience In/Out Choice (per 500ft^2^)	The average weighted (by percent time) size of all environments in which an elephant spent time where there is a choice of indoors or outdoors
	Joint Abnormalities (ref: absence)	Presence or absence of gait change, limb deformity, joint heat or swelling noted from muscoskeletal exam
Foot Health^6^	Percent Time In/Out Choice	Sum of monthly percent time spent housed in a social group of one
	Space Experience Total Night (per 500 ft^2^)	The average weighted (by percent time) size of all environments in which an elephant spent time at night
Walking Distance^7^	Mean Daily Walking Distance	Mean outdoor daily walking distance measured by anklets equipped with GPS data loggers
	Social Group Contact	Maximum number of unique social groups focal animal is part of
	Feeding Predictability (ref: unpredictable)	The predictability of feeding times; categorical where 1 is predictable, 2 is semi-predictable, and 3 is unpredictable
	Space Experience Total Night (per 500 ft^2^)	The average weighted (by percent time) size of all environments in which an elephant spent time in outdoor environments only
Serum Cortisol^8^	Mean Serum Cortisol	Mean of 24 blood samples taken bi-weekly for 1 year
	Keeper Attitude: Positive Opinions of Elephants	Composite scores (averaged by zoo) of keepers’ opinions of elephants: elephants are playful, like to be trained, like change, are trusting, affectionate, and bond to keepers
	Keeper Attitude: Keeper as Herdmate	Composite scores (averaged by zoo) of keepers’ perceptions that they are accepted by elephants as part of the herd, elephants are interested in the keepers, keepers connect verbally with elephants, keepers have bonds with elephants
	Latitude of Zoo	Angular distance of a zoo’s location north of the equator
	Elephant Positive Behaviors	Composite scores (from keeper ratings) for affiliative/friendly behaviors, food sharing, solo play, wallowing
	Elephant Interacts with Public	Composite scores (from keeper ratings) for elephant watches and initiates interactions with zoo visitors

^1^Identified in published studies of the EWP:

^2^Brown et al. [4];

^3^Morfeld et al. [7];

^4^Greco et al. [43];

^5^Holgate et al. [8];

^6^Miller et al. [9];

^7^Holgate et al. [6];

^8^Carlstead et al. [18].

Generalized Linear Mixed Models (GLMM) were used to determine *Species* and *Season* effects on mean FGMs, and *Species* and *Sex* effects on mean and CV of FGMs. *Zoo* was treated as a random effect to account for clustering of elephants by facility.

Mean FGM concentrations for elephants of each species, and CV of FGMs for both species combined, were fitted in regression models using Generalized Estimating Equations (GEE), which allow for the individual elephant to be used as the unit of analysis, accounts for clustering of individuals within zoos, and focuses on population-averaged effects [45]. GEE also allows for weaker distributional assumptions than mixed models, and was the technique used in previous EWP reports [4–9, 18, 42–43]. The model included repeated measures of FGMs by *Season*. Zoos were treated as random effects and an independent correlation structure was specified. We built multi-variable regression models by first assessing individual predictors at the univariate level and then at the bivariate level with each demographic variable (*Species*, *Age*, *Sex)* as potential confounding variables. Confounding variables (those that altered the beta values of input variables by more than 10% during bivariate analysis) were included in all models as necessary. Any variables that predicted FGM mean or CV (P < 0.15) following the univariate and bivariate assessments were retained for evaluation in the hierarchical model building process. The model building process proceeded using the forward selection approach [46]. Models reaching the multi-collinearity criteria, as defined by a variance inflation factor of greater than 10 and a condition index of greater than 30, were not considered for further analysis [46]. The forward selection of variables was continued until the addition of variables no longer resulted in significant models. Interactions were assessed during the final model building stage and the final model was selected based on quasi-likelihood under the independence model criterion (QIC) values [47] and parameter estimates of explanatory variables. With the exception of the univariate stage of the model building process where P < 0.15 was considered significant for continued analyses, P < 0.05 was considered statistically significant in the remainder of the model building stages. For other analyses, unless otherwise indicated, differences were considered significant at P < 0.05. All analyses were conducted using IBM SPSS Statistics Version 25, IBM Corp., Armonk, NY, USA.

## Results

The elephant study population ranged in age from 0 to 64 years (mean age: Asian, 34.3 ±1.5; African, 27.7 ±1.1 years). Table 2 presents seasonal mean FGM concentrations for each species. Overall FGM concentrations were higher in Asian (124.4 ± 4.9 ng/g) than African (97.7 ± 3.0 ng/g) elephants. There was a significant main effect of species (F = 27.86, df1,2 = 1,927, P = 0.000), but not season (F = 1.30, df1,2 = 3,927, P = 0.0001). In all seasons, Asian elephants had higher mean concentrations than Africans.

**Table 2:** Mean (± SEM) and minimum-maximum seasonal fecal glucocorticoid metabolite (FGM) concentrations in Asian (n = 106) and African (n = 131) elephants in North American zoos that participated in the Elephant Welfare Project.

Season	Asian Elephants			African Elephants		
	FGM Mean (ng/g)	Min	Max	FGM Mean (ng/g)	Min	Max
Winter (Jan-Mar)	146.91 ± 5.01^a^	43.41	317.67	108.48 ± 3.03^b^	31.83	222.49
Spring (Apr-Jun)	156.83 ± 5.04^a^	57.78	286.74	107.22 ± 3.01^b^	37.56	266.17
Summer (Jul-Sep)	146.29 ± 4.27^a^	49.74	324.18	105.04 ± 2.94^b^	28.81	229.71
Fall (Oct-Dec)	147.78 ± 5.13^a^	37.82	310.56	110.01 ± 3.08^b^	26.78	292.43

^a,b^Seasonal differences between species are significant (P < 0.05).

Mean and average variability (CV) of FGMs was calculated for the entire year and is given for each species and sex separately in Table 3. GLMM analysis found significant differences in mean FGM for *Species* (F = 8.496, df1,2 = 1,236, P = 0.004), but not for *Sex* (F = 0.124, df1,2 = 1,236, P = 0.726, Table 3). For FGM CV, which is a normalized calculation, there were no significant effects of *Species* (F = 0.004, df1,2 = 1,236, P = 0.950) or *Sex* (F = 0.891, df1,2 = 1,236, P = 0.346). Therefore, mean FGMs were analyzed separately for each species, whereas FGM CVs were analyzed for both species combined.

**Table 3:** Mean (± SEM) fecal glucocorticoid metabolite (FGM) concentrations and coefficient of variation (CV) for male and female Asian and African elephants in North American zoos that participated in the Elephant Welfare Project.

	Asian Elephants		African Elephants	
	Male = 21	Female = 85	Male = 27	Female = 104
**Mean FGM (ng/ml)**	121.55 ± 8.69^a^	125.47 ± 4.87^a^	99.61 ± 5.70^b^	97.72 ± 3.14^b^
**Mean FGM CV**	31.53 ± 1.49^a^	32.44 ± 1.28^a^	35.22 ± 2.55^a^	33.17 ± 1.18^a^

^a,b^Sex differences within species are significant (P < 0.05).

Descriptive statistics for independent variables are presented for each species in Table 4.

**Table 4:** Descriptive statistics (mean, SEM, minimum, maximum) for independent variables of Asian and African elephants in North American zoos that participated in the Elephant Welfare Project.

	Asian Elephants					African Elephants				
	N	Mean	SEM	Min	Max	N	Mean	SEM	Min	Max
Fecal Glucocorticoid Metabolites (ng/g)—Mean	106	124.69	4.26	59.69	282.88	131	98.11	2.75	40.56	211.34
Fecal Glucocorticoid Metabolites (ng/g)—CV	106	32.26	1.07	9.78	71.24	131	33.59	1.070	15.20	92.59
Percent Time in Mixed Sex Herds	106	12.46	2.969	0.00	100.00	131	23.31	3.200	0.00	100.00
Enrichment Diversity	93	2.91	0.015	2.54	3.16	129	2.83	0.014	2.54	3.26
Alternate Feeding Methods	100	0.49	0.022	0.08	0.92	131	0.38	0.019	0.08	0.91
Social Group Contact	106	2.70	0.200	1.00	11.00	131	4.94	0.618	1.00	30.00
Walking, Hours/Week	88	2.58	0.186	1.00	7.00	129	1.92	0.130	1.00	7.00
Feeding Diversity	95	1.37	0.032	0.31	1.78	129	1.38	0.018	0.98	1.79
Sex (ref: male)	106	0.80	0.039	0.00	1.00	131	0.79	0.035	0.00	1.00
Percent Time Managed	89	55.42	2.035	20.00	91.00	129	49.34	1.640	13.00	100.00
Percent Time with Juveniles	106	18.63	3.413	0.00	100.00	131	22.78	3.310	0.00	100.00
Percent Time Housed Separately	106	32.96	3.817	0.00	100.00	131	21.15	2.590	0.00	100.00
Transfers	106	2.69	0.204	0.00	10.00	129	2.68	0.162	0.00	10.00
Percent Time In/Out Choice	106	15.74	2.157	0.00	77.67	131	17.30	1.820	0.00	89.82
Social Experience	106	2.17	0.106	1.00	4.93	131	3.14	0.218	1.00	11.22
Recumbence Rate	25	8.02	0.752	0.00	19.72	38	5.34	0.452	0.05	9.17
Percent Time on Hard Substrate	106	9.69	1.260	0.00	51.80	131	13.13	1.080	0.00	50.00
Percent Time Soft Substrate	106	10.82	1.228	0.00	55.90	131	10.61	1.260	0.00	58.30
Space Experience Outdoor Night (per 500 ft^2^)	106	34.60	3.903	0.00	187.39	131	70.75	8.910	0.00	574.28
Space Experience In/Out Choice (per 500 ft^2^)	106	19.36	2.177	0.00	92.13	131	38.35	5.560	0.00	312.74
Joint Abnormalities (ref: absence)	98	0.33	0.048	0.00	1.00	94	0.23	0.044	0.00	1.00
Space Experience Total Night (per 500 ft^2^)	106	27.64	2.760	1.09	147.05	131	56.25	6.920	0.88	419.14
Age of Elephant	106	34.84	1.459	1.00	64.00	131	27.85	1.060	0.00	52.00
Mean Daily Walking Distance	26	5.31	0.629	1.21	17.26	34	5.42	0.260	2.19	9.71
Feeding Predictability (ref: unpredictable)	95	2.16	0.066	1.00	3.00	129	1.93	0.050	1.00	3.00
Mean Serum Cortisol	98	17.83	0.748	5.96	40.02	115	17.95	0.583	5.87	37.26
Keeper Attitude: Positive Opinions of Elephants	84	3.68	0.053	1.59	4.40	106	3.65	0.050	2.77	5.37
Keeper Attitude: Keeper as Herdmate	84	3.02	0.073	2.00	4.48	106	2.65	0.054	1.41	4.03
Latitude of Zoo	103	35.81	0.567	21.00	47.00	131	35.60	0.414	26.00	47.00
Elephant Positive Behaviors	67	4.45	0.128	1.53	6.31	93	4.67	0.080	2.21	6.42
Elephant Interacts with Public	67	2.48	0.107	0.98	5.68	93	2.40	0.082	0.83	5.16

For Asian and African elephants separately, univariate linear regressions of independent variables with mean FGM concentrations are shown in Table 5. For Asians, significant negative associations (i.e., lower FGMs) were observed for *Enrichment Diversity*, *Walking (hr/week)*, *Percent Time Managed by Staff*, *Experience Outdoors at Night*, *Space Experience with In/Out Choice*, *Total Space Experienced at Night*, *Mean Daily Walking Distance* and *Latitude of Zoo*. Positive associations (i.e., higher FGMs) were associated with *Percent Time Housed Separately*, *Recumbent Rate*, *Joint Abnormalities*, *Serum Cortisol* and *Keeper as Herdmate*. For Africans, significant negative regressions with mean FGMs were with *Percent Time Managed* (as with Asians), and *Percent Time with In/Out Choice*, and additionally with *Keeper as Herdmate*. Positive associations were with *Percent Time in Mixed Sex Herds*, *Social Experience*, *Social Group Contact*, *Feeding Predictability*, *Latitude of Zoo*, *Mean Daily Walking Distance*, and all three *Space Experience* variables. Therefore, African FGMs were positively associated with three social variables and only one individual variable (*Mean Daily Walking Distance*), whereas FGMs in Asians were positively associated with only one social variable (*Percent Time Housed Separately*) and four individual variables. Lastly, there was no age effect on FGM for either species.

**Table 5:** Univariate linear regressions of 12-month mean fecal glucocorticoid metabolite concentrations in Asian and African elephants in North American zoos and previously published risk factors (independent variables) from the Elephant Welfare Project. Variables at P<0.15 were considered significant for inclusion in the multi-variable analyses, and are **bolded**.

	Asian Elephants				African Elephants			
Independent Variable	N	Estimate	SEM	*P* value	N	Estimate	SEM	*P* value
Percent Time in Mixed Sex Herds (*unpub*.)	106	-0.065	0.140	0.646	**131**	**0.211**	**0.073**	**0.005**
Enrichment Diversity	**93**	**-58.746**	**31.058**	**0.062**	129	14.139	16.989	0.407
Alternate Feeding Methods	100	16.049	20.348	0.432	131	13.994	12.529	0.266
Social Group Contact	106	-0.312	2.088	0.882	**131**	**0.944**	**0.383**	**0.015**
Walking, Hours/Week	**88**	**-4.796**	**2.673**	**0.076**	129	-2.274	1.864	0.225
Feeding Diversity	95	-10.397	14.750	0.483	129	8.369	13.265	0.529
Sex (ref: male)	106	3.971	10.721	0.712	133	-1.543	6.804	0.821
Percent Time Managed	**89**	**-0.545**	**0.253**	**0.034**	**128**	**-0.284**	**0.149**	**0.060**
Percent Time with Juveniles	106	-0.043	0.122	0.726	131	0.079	0.073	0.283
Percent Time Housed Separately	**106**	**0.174**	**0.108**	**0.109**	131	0.023	0.093	0.804
Transfers	106	-0.964	2.040	0.637	131	-0.852	1.479	0.566
Percent Time In/Out Choice	106	-0.074	0.188	0.695	**131**	**-0.285**	**0.166**	**0.088**
Social Experience	106	-5.197	3.918	0.188	**131**	**2.342**	**1.089**	**0.033**
Recumbence Rate	**25**	**4.949**	**2.200**	**0.034**	38	0.908	1.639	0.583
Percent Time on Hard Substrate	106	0.725	0.323	0.027	131	0.132	0.223	0.556
Percent Time Soft Substrate	106	-0.115	0.340	0.735	131	0.229	0.190	0.229
Space Experience Outdoor Night (per 500 ft^2^)	**106**	**-0.187**	**0.105**	**0.080**	**131**	**0.073**	**0.026**	**0.006**
Space Experience In/Out Choice (per 500 ft^2^)	**106**	**-0.333**	**0.189**	**0.081**	**131**	**0.110**	**0.042**	**0.010**
Joint Abnormalities (ref: absence)	**95**	**20.198**	**7.470**	**0.008**	96	0.298	7.660	0.969
Space Experience Total Night (per 500 ft^2^)	**106**	**-0.282**	**0.149**	**0.060**	**131**	**0.111**	**0.033**	**0.001**
Age of Elephant	106	0.261	0.285	0.361	133	-0.278	0.227	0.222
Mean Daily Walking Distance	**26**	**-5.144**	**2.380**	**0.041**	**34**	**6.428**	**3.264**	**0.058**
Feeding Predictability (ref: unpredictable)	95	0.642	7.087	0.928	**129**	**6.221**	**4.167**	**0.138**
Mean Serum Cortisol	**98**	**1.208**	**0.591**	**0.024**	117	0.196	0.475	0.680
Keeper Attitude: Positive Opinions of Elephants	84	6.814	10.654	0.524	108	-3.814	4.838	0.432
Keeper Attitude: Keeper as Herdmate	**84**	**16.663**	**7.625**	**0.032**	**108**	**-10.227**	**4.683**	**0.031**
Latitude of Zoo	**106**	**-1.153**	**0.665**	**0.086**	**133**	**1.659**	**0.563**	**0.004**
Elephant Positive Behaviors	67	-5.672	4.667	0.229	93	-0.505	3.728	0.893
Elephant Interacts with Public	67	-0.212	5.644	0.970	93	0.503	3.639	0.890

Multivariable analyses required the exclusion of *Mean Daily Walking Distance* and *Recumbent Rate* because these variables were measured in only a sub-set of the elephants. Also, *Social Experience* was highly correlated (r = 0.899) with *Social Group Contact* and so was not included in the multivariable model building process due to collinearity problems. The final models are given in Table 6 for Asian and Table 7 for African elephants.

**Table 6:** Multi-variable model of seasonal fecal glucocorticoid metabolite concentrations for Asian elephants (n = 106) in North American zoos that participated in the Elephant Welfare Project^1^. Significant variables are **bolded**.

Variable	Beta Estimate	SEM	*P* value
Intercept	118.69	23.60	0.001
*Season*: Winter (Jan-Mar)	-2.43	24.92	0.922
*Season*: Spring (Apr-Jun)	-42.59	24.01	0.076
*Season*: Summer (Jul-Sep)	-10.91	21.21	0.606
*Season*: Fall (Oct-Dec) (ref)	0		
Sex: Female	-3.15	6.83	0.644
Sex: Male (ref)	0		
Age of Elephant	0.34	0.22	0.128
Joint Health: No Abnormalities	**-21.14**	**8.58**	**0.014**
Joint Health: Abnormalities (ref)	0		
Space Experience In/Out Choice (per 500 ft^2^)	**-0.41**	**0.13**	**0.003**
*Season*: Winter*Latitude of Zoo	0.61	0.66	0.350
*Season*: Spring*Latitude of Zoo	**1.81**	**0.77**	**0.019**
*Season*: Summer*Latitude of Zoo	0.66	0.62	0.288
*Season*: Fall*Latitude of Zoo (ref)	0.39	0.55	0.473

^1^*Age* is a confounder for *Sex* and *Latitude of Zoo*.

**Table 7:** Multi-variable model of seasonal fecal glucocorticoid metabolite concentrations for African elephants (n = 131) in North American zoos that participated in the Elephant Welfare Project^1^. Significant variables are **bolded**.

	Beta Estimate	SEM	*P* value
Intercept	16.67	26.24	0.525
*Season*: Winter (Jan-Mar)	-3.79	2.94	0.197
*Season*: Spring (Apr-Jun)	-1.10	3.03	0.716
*Season*: Summer (Jul-Sep)	-1.71	2.80	0.541
*Season*: Fall (Oct-Dec) (ref)	0		
Sex: Female	-5.53	6.69	0.409
Sex: Male (ref)	0		
Age	-0.10	0.28	0.719
Percent Time Managed	**-0.27**	**0.13**	**0.045**
Latitude of Zoo	**2.62**	**0.58**	**0.001**
Percent Time in Mixed-Sex Herds	**0.19**	**0.09**	**0.039**
Space Experience Outside at Night (per 500 ft^2^)	**0.06**	**0.02**	**0.004**
Percent Time In/Out choice	**-0.20**	**0.09**	**0.032**

^1^*Age* of elephant is a confounder of *Percent Time Managed* and *Latitude of Zoo*. *Latitude of Zoo* was a confounder of *Percent Time in Mixed-Sex Herds* and *Space Experience Outside at Night*.

The initial, best multi-variable model for Asian elephant FGMs showed trending effects for *Season*: *Spring* and *Latitude of Zoo* (P = 0.076 and 0.051, respectively), so Season*Latitude of Zoo was added as an interaction term in the model. The rationale for this was that the degree of climatological change between seasons is a function of how far north the zoo lies. With the interaction term added to the model, *Latitude of Zoo* was no longer significant as a main effect and was dropped from the model (Table 6). The interaction factor was a significant risk factor for higher FGM only in the spring season at higher latitudes. When all other independent variables are held constant, an increase of one degree in *Latitude of Zoo* corresponds to a 1.81 ng/g increase in FGM during April—June. For Asian elephants, risk factors for higher FGMs were *Joint Abnormalities* and limited *Space Experience with In/Out Choice*. Our analysis found that, when all other independent variables are held constant, the absence of *Joint Abnormalities* decreases FGM by 21.14 ng/g, and for every 5000 ft^2^ increase in *Space Experience with In/Out Choice* there is a 4.1 ng/g decrease in FGM.

The multivariable model for African elephant FGMs also demonstrated effects of *Latitude of Zoo* on FGM, but no seasonal effects (Table 6). As latitude increases by one degree, FGMs increase by 2.67 ng/g. There were four additional risk factors in the multivariable model: *Percent Time In/Out Choice*, and *Percent Time Managed* by staff. For every 10% increase in *Percent Time In/Out Choice* there is a 2.00 ng/g decrease in FGM. Similarly, a 10% increase *Percent Time Managed* decreases FGMs by 2.70 ng/g. By contrast, *Percent Time in Mixed-Sex Groups* and *Space Experience Outdoors at Night* increase FGMs: a 10% increase in time produces a 1.90 ng/g increase, and a 5000 ft^2^ increase in space experience produces a 0.60 ng/g in FGMs.

Table 8 presents univariate regressions of the independent variables and FGM CV. Associated with lower FGM variability were *Enrichment Diversity*, *Social Group Contact* and *Social Experience*, *Percent Time with Juveniles*, both *Space Experience at Night* variables, *Mean Daily Walking Distance*, *Feeding Predictability* and *Latitude of Zoo*. The variable associated with increased variability was *Percent Time with In/Out Choice*.

**Table 8:** Univariate linear regressions between CV of fecal glucocorticoid metabolite concentrations and previously published risk factors (independent variables) for Asian and African elephants in North American zoos that participated in the Elephant Welfare Project. Variables at P<0.15 were considered significant for inclusion in the multi-variable analyses, and are **bolded**.

Independent variable	N	Beta	SE	P value
Percent Time in Mixed Sex Herds (unpublished)	237	-0.015	0.022	0.507
**Enrichment Diversity**	**222**	**-14.524**	**4.566**	**0.002**
Alternate Feeding Methods	231	-2.216	3.421	0.518
**Social Group Contact**	**237**	**-0.451**	**0.135**	**0.001**
Walking (14 or more hours per week)	217	-0.342	0.470	0.468
Feeding Diversity	224	-1.395	3.008	0.643
Sex (ref: male)	237	-0.790	1.894	0.677
Percent Time Managed	218	0.022	0.040	0.580
**Percent Time with Juveniles**	**237**	**-0.042**	**0.021**	**0.044**
Percent Time Housed Separately	237	-0.004	0.022	0.858
Transfers	237	0.411	0.363	0.260
**Percent Time In/Out Choice**	**237**	**0.102**	**0.035**	**0.004**
**Social Experience**	**237**	**-0.830**	**0.370**	**0.026**
Recumbence Rate	63	0.229	0.465	0.625
> 0 Percent Time on Hard Substrate	237	-0.012	0.060	0.838
> 0 Percent Time Soft Substrate	237	0.039	0.056	0.486
**Space Experience Outdoors Night**	**237**	**-0.016**	**0.009**	**0.076**
Space Experience In/Out Choice (per 500 ft^2^)	237	-0.016	0.015	0.304
Joint Health: Absence or presence of joint abnormalities	194	0.952	1.940	0.624
**Space Experience Total Night (per 500 ft**^**2**^**)**	**237**	**-0.020**	**0.012**	**0.099**
Age of Elephant	237	0.039	0.055	0.477
**Mean Daily Walking Distance**	**60**	**-1.832**	**0.640**	**0.041**
**Feeding Predictability (ref: Unpredictable)**	**224**	**-2.564**	**1.145**	**0.026**
Mean Serum Cortisol	215	-0.023	0.116	0.844
Keeper Attitude: Positive Opinions of Elephants	192	-1.561	1.641	0.343
Keeper Attitude: Keeper as Herdmate	192	1.373	1.356	0.312
**Latitude of Zoo**	**237**	**-0.358**	**0.146**	**0.015**
Elephant Positive Behaviors	160	1.363	1.010	0.179
Elephant Interacts with Public	160	-0.822	1.106	0.458
Species (ref = 2, Asian)	237	-1.282	1.52	0.402

The multivariable model for FGM CV (Table 9) indicates that *Percent Time In/Out Choice* increases FGM variability: when other variables are held constant, for each 10% increase in time there is a 0.9% increase in CV of FGM. *Enrichment Diversity* and *Social Group Contact* both decreased variability. Each 1.0 increase in the Shannon Diversity Index of enrichment is associated with a 13.4% decrease in the CV of FGMs, and each additional *Social Group Contact* results in a 0.5% decrease. *Species* confounds *Enrichment Diversity* and *Social Group Contact* due to Asian elephants receiving, on average, slightly more enrichment than Africans (see Table 4), and Africans having contact with more social groups than Asians (Table 4), primarily because Africans are kept more often in larger groups.

**Table 9:** Multi-variable model of CV of fecal glucocorticoid metabolite concentrations for Asian (n = 106) and African (n = 131) elephants in North American zoos that participated in the Elephant Welfare Project^1^. Significant variables are **bolded**.

Independent variable	Beta	SEM	P value
Species^1^ (ref: Asian)	0.925	1.3855	0.504
Sex (ref: female)	0.828	1.7213	0.630
Age	-0.050	0.0698	0.477
Percent Time In/Out Choice	**0.090**	**0.0390**	**0.021**
Enrichment Diversity	**-13.430**	**4.1904**	**0.001**
Social Group Contact	**-0.516**	**0.0983**	**0.000**

^1^Species is a confounder of *Social Group Contact* and *Enrichment Diversity*.

**Fig 1:**
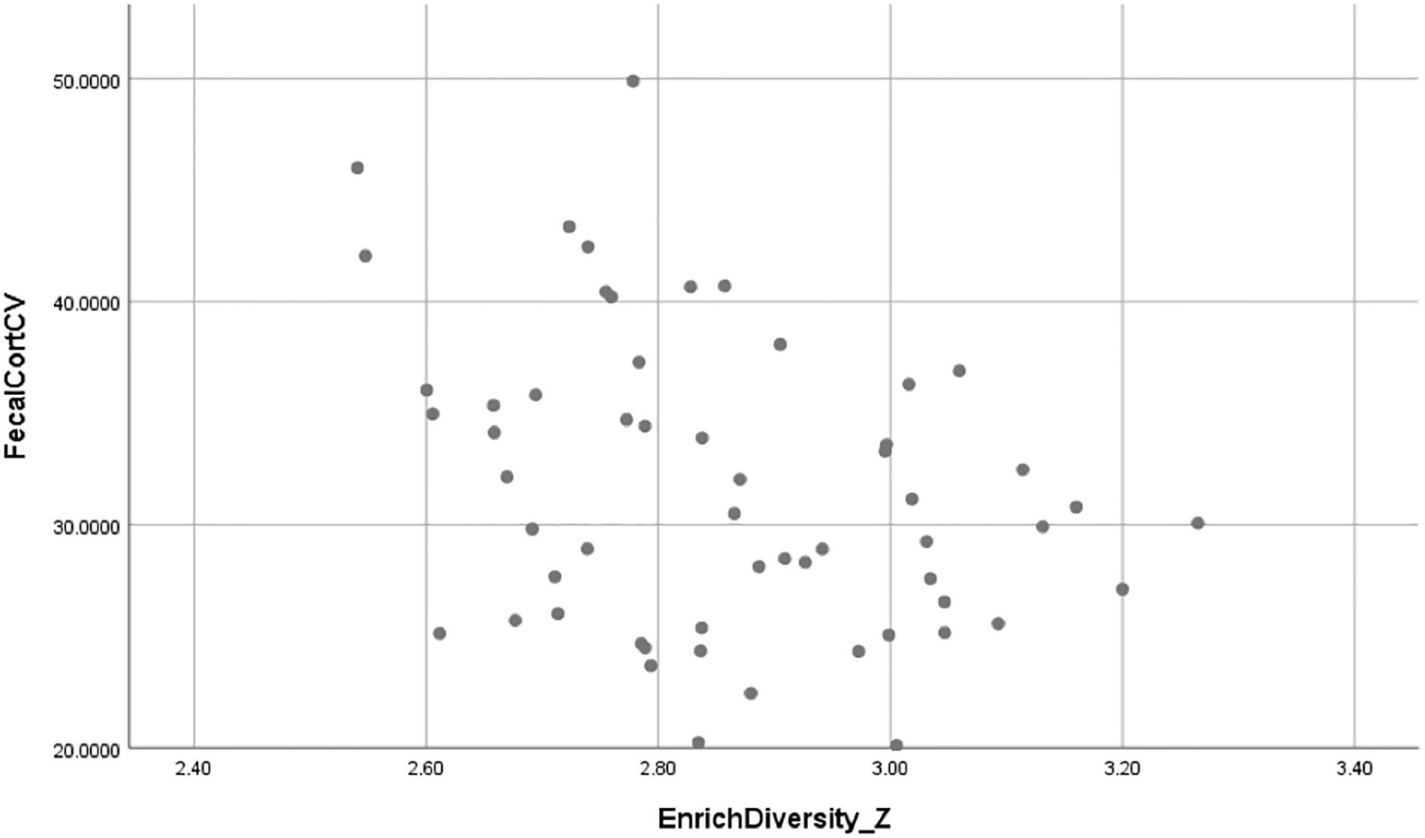
Correlation between zoos’ enrichment diversity scores and mean coefficient of variation (CV) of fecal glucocorticoid metabolite concentrations at zoos (r = -0.339, n = 57, *P* = 0.010).

Because *Enrichment Diversity* was calculated on a zoo-level, Fig 1 shows the correlation between a zoo’s enrichment diversity score and the average FGM CV of the elephants at a zoo.

## Discussion

Epidemiological analyses of the EWP data point to a number of individual, social, housing and management factors that might affect adrenal activity in the zoo-housed elephant population in North America. A higher risk of elevated FGM concentrations was found for Asian elephants with joint abnormalities, and African elephants housed in mixed-sex herds, whereas all elephants housed in northern latitudes had an increased risk of higher FGM in the spring (Asians) or all seasons (Africans). More importantly, the results point to management factors that decrease FGMs in both species: having choice of being indoors and out, and management interactions with staff (Africans). The variability in FGM concentrations (CV) was reduced by enrichment and social groupings, and increased by having a choice of indoor and outdoor spaces. Interestingly, univariate analyses indicated that walking distance and all three space experience variables were negatively correlated to FGM in Asian elephants, but positively associated in African elephants. These patterns suggest there are species differences in how housing space is experienced, which may indicate that species-specific management protocols are needed.

Having the choice to be indoors or out appears to decrease adrenal activity for both species, as indicated by significant negative associations between mean FGM concentrations and the independent variables *Space Experience with In/Out Choice* (Asians) and *Percent Time with In/Out Choice* (Africans). Greco et al. [43] also identified *Percent Time with In/Out Choice* as a factor that reduced the frequency of nighttime stereotypy in the current population. Choice is generally beneficial to the welfare of captive animals because it increases an animal’s perceived control over its environment [48] and being given a choice of moving between indoor and outdoor areas at will has been associated with reduced stereotypic behaviors in polar bears [49], Asian elephants [50], and giant pandas [51]. For Asian elephants, those with joint problems had higher FGMs than those that did not, presumably due to pain. This could be the result of spending more time on hard surfaces and being older on average than African elephants in this population, because *Time on Hard Surfaces* and *Age* are both risk factors for joint problems [9].

*Latitude of Zoo* was a risk factor for higher FGMs in African elephants, increasing as a zoo’s location was more northwards. For Asians, this effect was only identified in the spring. Carlstead et al. [18] also found that *Latitude of Zoo* was a predictor of higher serum cortisol in this same population of Asian elephants. There are a variety of elephant management modifications that take place as seasons change, such as elephants spending more time confined inside or outside, with potential changes in social density or social contact that could account for increased social stress [52]. Higher glucocorticoids have been reported during colder seasons among small numbers of zoo-housed Asian [53] and African [54] elephants. In Thailand, mean FGM concentrations were ~28% higher in winter compared to the summer and rainy seasons, and were negatively associated with temperature and rainfall, but not humidity [55]. The need for more energy to maintain optimum body temperature and ensure survival in cooler temperatures could be related to this finding.

There were three other risk factors identified for African FGMs. First, *Percent Time Managed* by staff reduces FGMs, and also reduces daytime stereotypies for both species [43]. In Asians, there was a significant univariate correlation between FGMs and *Percent Time Managed*, but it did not make it into the multivariable model. Therefore, stress in African elephants, as indicated by higher FGM concentrations and higher rates of stereotypy in the day time, may be due to insufficient time spent in interactions with staff (i.e. cleaning and grooming, feeding, exercising and training). Positive interactions with keeper staff have been shown to be predictors of lower serum cortisol concentrations for both species [18]. The evidence points strongly to interactions with staff being stress-reducing for elephants. Second, *Percent Time in Mixed-Sex Herds* was associated with increased FGMs, possibly related to having bulls for breeding, a natural stressor. The third risk factor for African FGMs was *Space Experience Outdoors at Night*. There is no obvious explanation for why having more outdoor space at night would be associated with increased adrenal activity. Perhaps there are more social interactions occurring under the cover of darkness, without keepers nearby, which for some elephants might be stressful or, alternatively, stimulating. Posta et al. [56] reported that two zoo-housed African elephants spent a greater portion of their time outdoors at night walking, while others report significant social behaviors occurring during the night with free access to indoor and outdoor areas [57,58]. Holdgate et al. [6] also found that a subset of elephants from this population had a greater *Mean Walking Distance* if they had a greater *Space Experience at Night*. Therefore, evidence suggests that outdoor space at night facilitates activity of African elephants, and increased activity could account for the slight increase in FGMs identified in the multi-variable model.

In assessments of FGM CVs, three risk factors were identified: *Percent Time In/Out Choice*, *Enrichment Diversity* and *Social Group Contact*. Having more choice of being indoors or outdoors was associated with a decrease in mean FGM in both species. Therefore, while the overall population effect of choice appears to be stress-reducing, it leads to slightly increased variability within individuals. We speculate that this may be due to movements of other elephants in the herd going in and out in an unpredictable manner. A given individual might benefit from having increased choice and control over its own situation, but it has no control over the whereabouts of other elephants, potentially resulting in more variable stress responses. Cochrem [59] points out that CV should be included in studies of GCs because the factors that account for within-individual variation and their adaptive significance, such as personality, coping styles, genetic or maternal influences, are little known for most species. For example, increased variability in FGMs was correlated with abnormal reproductive function, higher rates of fighting, and institutional mortality rates in rhinoceros [60], leading to the conclusion that the variability of FGMs is a valuable measure of stress responsiveness that may have biological costs to the animal. The subject of individual variation in GC responses to stressors has included investigations of differences in coping styles and disease susceptibility [61]. A better understanding of inter- and intra-individual variation in hypothalamo-pituitary-adrenal activity would be beneficial to our use of GCs as a welfare measure as suggested by Edwards et al. [42].

*Enrichment Diversity* was strongly associated with a reduction in CV of FGMs, but not with mean FGMs, suggesting that having multiple enrichment options functions to moderate adrenal reactivity of individuals. Brown et al. [4] found enrichment diversity to be positively correlated with reproductive health in African females of the EWP, both in terms of reduced acyclicity and normalization of prolactin secretion, and our results support enrichment as an important management factor for zoo elephant welfare. All elephants of the EWP received some form of enrichment at their zoo, and the frequency with which different enrichments were provided was found to impact the variability of FGMs within, but not between individuals. An analogous experiment with mice found that housing in enrichment diverse “calming” environments, consisting of a large cage with a cardboard nest box, paper nesting material, and a tube, exhibited significant and lasting reductions over time in FGM levels compared to mice housed in less enriched, standard caging [62]. In our study, *Enrichment Diversity* scores were derived from surveys of zoo managers providing the percentage of days their elephants had access to 30 different types of enrichment items, ranging from exhibit features such as sand or dirt piles, mud wallows, pools, logs, scratching posts and sprinklers, to the provision of manipulatable objects such as balls, tires and hanging objects, to feeding items such as browse and treat boxes/bags, and scents, music and problem-solving tasks [5]. We found the zoo average FGM CVs to be negatively correlated with the frequency of only three of the 30 enrichment types: problem-solving (r = -0.348, n = 57, p = 0.007), hanging objects (r = -0.261, p = 0.048) and scratching posts (r = -0.340. p = 0.009); three enrichments that intensely engage elephants. All evidence together strongly suggests that enrichment has a “calming” effect on stress responses of elephants, most likely by providing additional behavioral options and/or cognitive opportunities to cope with their daily lives.

Last, being a member of more social groups (*Social Group Contact*) also was associated with lower variability in FGMs. Therefore, being a familiar and accepted member of multiple social groups may also stabilize activity of the adrenal cortex in a manner similar to *Enrichment Diversity*, effectively increasing social enrichment diversity, a clear benefit for elephant welfare.

## Conclusions

Results elucidate species differences in FGM concentrations of elephants in relation to a variety of zoo environments. A stress-related welfare problem was identified among Asian elephants with joint health problems. African elephants appear to be more responsive to social stressors than Asians, which fits with their natural history. African elephants form complex, multi-tiered social groups that are important to survival, whereas Asian herds are smaller and bonds are more fluid [63]. One factor that reduced FGMs for both species was more time being managed, suggesting time spent with keepers has a positive effect. More time being managed also was associated with reduced stereotypy [43]. Finally having diverse enrichment options and contact with multiple social groups also appears to be calming for elephants, reducing intra-individual variability in FGMs. Together, all evidence points to the beneficial effects of diverse enrichment opportunities, including cognitive enrichment for zoo-housed elephants. We conclude that there are many avenues for further research on stress in zoo-housed elephants, and monitoring FGMs longitudinally is a proven non-invasive method for determining factors contributing to adrenal function, stress and coping responses in elephants. The species differences in FGM responses to zoo factors suggests that a one-size-fits-all management strategy may not be appropriate, and that more species-specific approaches to husbandry are needed.
